# Genomic Epidemiology of CC30 Methicillin-Resistant Staphylococcus aureus Strains from Argentina Reveals Four Major Clades with Distinctive Genetic Features

**DOI:** 10.1128/mSphere.01297-20

**Published:** 2021-03-10

**Authors:** Sabrina Di Gregorio, María Sol Haim, Jesús Vielma Vallenilla, Victoria Cohen, Lucía Rago, Lucía Gulone, David M. Aanensen, Silvia Argimón, Marta Mollerach

**Affiliations:** a Universidad de Buenos Aires, Facultad de Farmacia y Bioquímica, Instituto de Investigaciones en Bacteriología y Virología Molecular (IBaViM), Buenos Aires, Argentina; b Consejo Nacional de Investigaciones Científicas y Técnicas (CONICET), Buenos Aires, Argentina; c The Centre for Genomic Pathogen Surveillance (CGPS), Wellcome Genome Campus, Hinxton, Cambridge, United Kingdom; d Big Data Institute, Li Ka Shing Centre for Health Information and Discovery, Nuffield Department of Medicine, University of Oxford, Oxford, United Kingdom; University of Nebraska Medical Center

**Keywords:** CC30, MRSA, *Staphylococcus aureus*, genomic epidemiology, whole-genome sequencing

## Abstract

Staphylococcus aureus clonal complex 30 (CC30) has given rise to epidemics worldwide and is one of the most prevalent lineages in Argentina, represented by sequence type 30 methicillin-resistant S. aureus SCC*mec* type IV (ST30-MRSA-IV). ST30-MRSA-IV has displaced previous prevalent clones in the country and demonstrated increased virulence. Despite the burden of infections caused by ST30-MRSA-IV both in hospitals and in communities in Argentina, no detailed genome-based characterization of this clone is available to date. In this study, we used whole-genome sequencing (WGS) to evaluate the genetic diversity, population structure, and genomic characteristics of 190 CC30-MRSA strains circulating in Argentina between 2004 and 2015. Phylogenetic analysis revealed the existence of 4 major clades: ARG-1 (CC30-MRSA-IVc-*spa* t012), ARG-2 (ST30-MRSA-IVc-*spa* t021 related), ARG-3 (ST30-MRSA-IVh/j-*spa* t021 and related), and ARG-4 (CC30-MRSA-IVc-*spa* t019 and related). The clades were characterized by different distributions of antimicrobial resistance determinants, virulence genes, and mobile genetic elements (MGEs). While ARG-1 and ARG-4 were related to global epidemic MRSA-16 (EMRSA-16) and South West Pacific (SWP) clones, respectively, ARG-3 was phylogenetically distinct from previously defined CC30 epidemic clones. ARG-4, the most prevalent and geographically disseminated in the collection (*N* = 164), was characterized by specific MGEs and chromosomal mutations that might have contributed to its virulence and success. To our knowledge, this is the first genomic epidemiology study of CC30-MRSA in Argentina, which will serve as baseline genomic data going forward to inform public health measures for infection prevention and control.

**IMPORTANCE** The rise in prevalence of community-associated methicillin-resistant Staphylococcus aureus (CA-MRSA) is of public health concern. In Argentina, several studies documented a shift in the epidemiology of CA-MRSA since 2009, with clonal complex 30 (CC30) and, in particular, sequence type 30 MRSA SCC*mec* type IV (ST30-MRSA-IV) replacing other clones both in communities and in hospitals and possibly displaying increased virulence. By sequencing the whole genomes of 190 CC30 MRSA isolates recovered from Argentina between 2005 and 2015, we showed that they represented a diverse population composed of 4 major clades. The predominant clade evolved from the South West Pacific clone but has acquired a distinct repertoire of mobile genetic elements, virulence genes, and chromosomal mutations that might play a role in its success. Our work is the first extensive genomic study of CC30 S. aureus in Argentina and will contribute not only to the development of genomic surveillance in the region but also to our understanding of the global epidemiology of this pathogen.

## INTRODUCTION

Methicillin-resistant Staphylococcus aureus (MRSA) is a relevant public health problem involving hospital-associated (HA-MRSA) and community-associated MRSA (CA-MRSA) strains (1). However, the definition of community and hospital strains is no longer a strict one, as CA-MRSA strains have been described as a cause of outbreaks in hospital settings, while HA-MRSA strains have been reported spreading in the community (2, 3).

Several MRSA clones have emerged worldwide, and the most prevalent ones are grouped into relatively few clonal complexes (CC) as defined by multilocus sequence typing (MLST [4]): CC1, CC5, CC8, CC22, CC30, CC45, CC59, and CC80 (5). Traditional molecular typing techniques for MRSA such as staphylococcal cassette chromosome *mec* (SCC*mec*) typing (6) and *spa* typing (7) also provide a nomenclature to describe relevant lineages.

Among them, CC30 is a major clonal complex that has caused a significant impact on global human health. Three pandemic lineages have been described within CC30 causing epidemic waves: methicillin-sensitive phage type 80/81 (sequence type 30 [ST30]-MSSA), epidemic MRSA-16 (EMRSA-16) (ST36 MRSA SCC*mec* type II [ST36-MRSA-II]), and the Southwest Pacific (SWP) clone (ST30-MRSA-IV). Phylogenomic analysis established that these three lineages evolved independently and revealed genetic determinants that might explain their virulence and niche adaptation. The differential acquisition of Panton-Valentine leukocidin (PVL) or toxic shock syndrome toxin (TSST) between CC30 lineages and mutations in *hla* and *agrC* leading to a virulence-attenuating effect for EMRSA-16 are among the most relevant genetic determinants described (8–10).

In Argentina, S. aureus is among the three most frequently isolated microorganisms in hospitalized adult patients (11). MRSA prevalence increased over the years, having maintained at approximately 50% in both hospital and community settings during 2010 to 2017 (12–14). The epidemiology of MRSA, however, changed over time, both in the hospital environment and in the community. Between 2004 and 2008, ST5-MRSA-IV-*spa* t311-PVL^+^ was reported as the most prevalent CA-MRSA clone both in adults and in children, while ST30-MRSA-IV-*spa* t019-PVL^+^ represented a minority clone in community settings (15, 16). Since 2009, however, different studies documented the rising prevalence of ST30-MRSA-IV. A nationwide multicenter study carried out during 2009 (12) and two multicenter studies between 2010 and 2011 (13, 17) described that the ST30-MRSA-IV-*spa* t019-PVL^+^ clone recovered mainly from adult patients had displaced the previously prevalent ST5-MRSA-IV-*spa* t311-PVL^+^ clone in community settings. Furthermore, several laboratory surveillance studies revealed the increasing prevalence of ST30-MRSA-IV-PVL^+^ strains recovered both from adults and from pediatric patients within the hospital environment (12, 18–20). The causes underlying the emergence and extinction of S. aureus clones over time are still unknown but are likely related to changes in lifestyles or antibiotic therapies (5, 21, 22).

PVL^+^ CA-MRSA strains were traditionally associated with skin and skin structure infections (SSSIs) (23). However, we previously reported that ST30-MRSA-IV-PVL^+^ was significantly associated with invasive infections in the community and had a more aggressive behavior in animal models of infection than ST5-MRSA-IV-PVL^+^ (24). Despite its clinical significance, the genetic diversity and virulence factors of ST30-MRSA-IV in Argentina have only been characterized with molecular techniques.

Whole-genome sequencing (WGS) has a greater discriminating power than molecular typing techniques (25) and can reveal the pathogen’s genetic traits underlying epidemiological changes (22). However, studies documenting the epidemiology of S. aureus in Latin America using genomics are scarce, and those available include a small number of isolates belonging to CC30 (26). This study used WGS to evaluate the genetic diversity of CC30 MRSA strains from Argentina spanning the period of the rise in prevalence of this clone (2004 to 2015), to characterize their repertoire of virulence factors, antimicrobial resistance mechanisms, and mobile genetic elements (MGEs), and to contextualize them with previously described global epidemic CC30 strains.

## RESULTS

### Population structure of CC30 MRSA from Argentina.

To characterize the population structure of CC30 MRSA, we studied 190 strains isolated in Argentina between 2004 and 2015 and previously characterized as CC30 MRSA in the laboratory. Most of the isolates were recovered from SSSIs (*N* = 118, 62.11%) followed by bacteremia (*N* = 43, 22.63%) and cystic fibrosis (CF)-related infections (*N* = 15, 7.89%), and from adult patients (*N* = 118, 62.11%). Only 2 isolates were recovered from healthy children. Whole-genome sequences showed that 186 strains belonged to ST30, and the remaining 4 strains belonged to 3 different STs that are single locus variants (SLVs) of ST30 and newly reported here (ST5999, ST6000, and ST6078) (see Tables S1 and S2 in the supplemental material).

10.1128/mSphere.01297-20.6 TABLE S1 Summary of source collections of isolates sequenced in this study. Download Table S1, XLSX file, 0.1 MB. Copyright © 2021 Di Gregorio et al. 2021 Di Gregorio et al. https://creativecommons.org/licenses/by/4.0/ This content is distributed under the terms of the Creative Commons Attribution 4.0 International license.

10.1128/mSphere.01297-20.7 TABLE S2 Metadata, QC, phenotypic and genotypic characteristics of CC30 methicillin-resistant S. aureus from Argentina. M, male; F, female; SSSI, skin and skin structure infection; R, resistant; S, susceptible. “yes,” presence of a genetic determinant; “no,” absence of a genetic determinant. Download Table S2, XLSX file, 0.2 MB. Copyright © 2021 Di Gregorio et al. 2021 Di Gregorio et al. https://creativecommons.org/licenses/by/4.0/ This content is distributed under the terms of the Creative Commons Attribution 4.0 International license.

**FIG 1:**
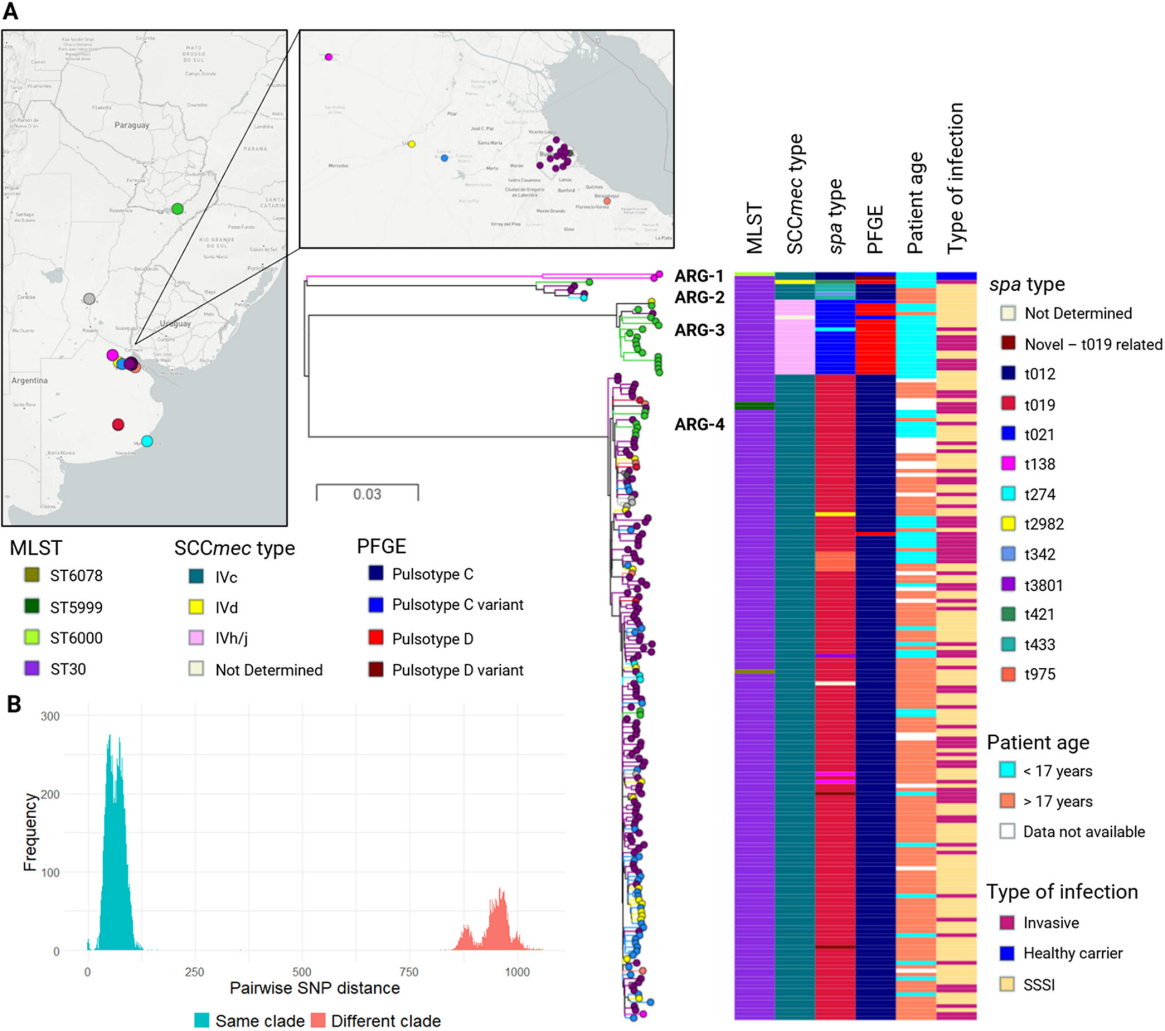
Phylogeny, molecular and demographic characteristics of Argentinean CC30 MRSA. (A) Midpoint-rooted phylogenetic tree inferred from 5,835 SNP sites obtained after mapping the genomes to the complete genome of strain ILRI_Eymole1/1 (ST30) and masking regions of recombination and MGEs. Tree branches and nodes are colored by city of isolation as indicated on the map. Scale bars represent the number of single nucleotide polymorphisms (SNPs) per variable site. The distributions of genotypes and demographic characteristics are shown as tree metadata blocks. Data are available at https://microreact.org/project/qpaxQtz-9/8ab43f7e. (B) Histogram of pairwise SNP differences between sequenced isolates. SNPs differences between isolates from the same or different clade are colored as described in the legend.

To further dissect the population, we identified 5,835 single nucleotide polymorphisms (SNPs) from whole-genome alignments against the reference genome of S. aureus strain ILRI_Eymole1/1 (ST30) after excluding regions of MGEs and regions of recombination. A phylogenetic tree inferred from whole-genome SNPs revealed the existence of four major clades with 100% bootstrap support each that largely coincided with the genotypic characterization (ARG-1 to ARG-4) (Fig. 1A). The distinct divergence between genomes belonging to different clades also supported the definition of ARG-1 to ARG-4 (Fig. 1B). ARG-1 contained only 2 strains from the city of San Antonio de Areco (CC30-MRSA-IVc-*spa* t012). ARG-2 included 5 strains from different locations, mainly with pulsed-field gel electrophoresis (PFGE) pulsotype C (ST30-MRSA-IVc-*spa* t021 and related). ARG-3 grouped 19 strains that were recovered mainly from children in the city of Posadas with PFGE pulsotype D (ST30-MRSA-IVh/j-*spa* t021 and related). ARG-4 is the largest clade, containing 164 strains with PFGE pulsotype C (CC30-MRSA-IVc-*spa* t019 and related). Strains within the dominant ARG-4 clade were recovered both from children and from adult patients, from all locations included in this study, and between 2005 and 2015, suggesting that this successful clade has persisted and disseminated across the territory. No association of major clades with the type of infection (SSSI or invasive) was observed (*P* > 0.05, chi-square test), but both strains recovered during a colonization study from healthy children clustered together in clade ARG-1, while a clinical strain from the same city was found in clade ARG-4. The combined phylogenetic and pangenome analyses revealed that the 4 major clades can be distinguished also by their accessory genome (see Fig. S1). These observations prompted us to investigate the genetic traits that may have contributed to the success of CC30 MRSA in Argentina, with an emphasis on clade ARG-4.

10.1128/mSphere.01297-20.1 FIG S1 Distribution of the accessory genome on the core genome phylogeny of 190 CC30 MRSA genomes from Argentina. Midpoint-rooted tree inferred from 5,302 core SNPs identified on Roary’s core genome alignment (2,316 core genes). The accessory genes are represented as blue blocks; the absence of a gene is represented in white. Download FIG S1, TIF file, 1.0 MB. Copyright © 2021 Di Gregorio et al. 2021 Di Gregorio et al. https://creativecommons.org/licenses/by/4.0/ This content is distributed under the terms of the Creative Commons Attribution 4.0 International license.

### Antimicrobial resistance of prevalent CC30 MRSA clades in Argentina.

First, we investigated the distribution of antimicrobial resistance (AMR) determinants and their association with MGEs. Clades ARG-1 to -4 were characterized by a specific repertoire of plasmid replicon (*rep*) genes, associated BlaZ types, and genes conferring resistance to arsenic and/or cadmium (Table 1; see also Fig. S2).

**TABLE 1:** Mobile genetic elements found in Argentinean CC30 MRSA major clades^*a*^

Genetic determinant	Mobile genetic elements			
	ARG-1 (*N* = 2)	ARG-2 (*N* = 5)	ARG-3 (*N* = 19)	ARG-4 (*N* = 164)
Plasmid *rep* and associated genes	*rep5*, *rep16*, *rep19*	*rep5*, *rep16*	*rep5*, *rep16*, *rep19*	*rep16*, *rep19*, *rep21*
	*blaZ* (BlaZ type A), *arsB*, *arsC*, *cadC*, *cadA*, *D2JAJ4*^*b*^	*blaZ* (BlaZ type C), *cadD*, *cadX*	*blaZ* (BlaZ type C), *arsB*, *arsC*, *cadD*, *cadX*	*blaZ* (BlaZ type A), *cadD*, *cadX*
Prophage *int* and associated genes	Sa3*int*, Sa5*int*	Sa1*int*, Sa2*int*, Sa3*int*	Sa2*int*, Sa3*int*	Sa2*int*
	*sea*, *sak*, *scn*, *chp*	*sek*, *seq*, *sea*, *sak*, *scn*, *chp*, *lukS/F-PV* (*H1a*)	*sak*, *scn*, *chp*, *lukS/F-PV* (*H1a*)	*lukS/F-PV* (*H2a*), *sak*, *scn*, *chp*^*c*^
SaPI *int* and associated genes	*int*SaPI2, *int*SaPI4, *tst-1*	Not detected	*int*SaPI2, *int*SaPI4	*int*SaPI3-like^*d*^
ICEs	ICE6013	Not detected	Not detected	Not detected^*e*^

a Table summarizes mobile genetic elements and associated genetic determinants of antimicrobial resistance and virulence. Only genes present in most (>80%) of the strains within each clade are shown.

b AMR genes *blaZ* (BlaZ type A), *arsB*, *arsC*, *cadC*, *cadA*, and *D2JAJ4* were present in clade ARG-1 but not found in association with any *rep* genes on the same contig for this clade.

c *sak*, *scn*, and *chp* were present in clade ARG-4 without an associated prophage integrase.

d *int*SaPI3-like integrase gene shared 96% identity with *int*SaPI3 from S. aureus COL reference sequence.

e ICE6013 interspersed in the phylogeny (*N* = 12).

10.1128/mSphere.01297-20.2 FIG S2 Genetic determinants of antimicrobial resistance and associated MGEs. Midpoint-rooted phylogenetic tree as in Fig. 1 but displaying tree branches and nodes colored by clade (ARG-1, turquoise; ARG-2, pink; ARG-3, light green; and ARG-4, dark blue). Colored horizontal blocks indicate the presence of a full/complete genetic determinant. Absence of a genetic determinant is colored in pale gray. Plasmidic *rep* genes are colored in purple. ICE6013 is colored in black. SCC*mec* types, BlaZ types, and AMR determinants are colored as described in the legend. MLS, macrolide, lincosamide, streptogramins; QAC, quaternary ammonium compounds. Data are available at https://microreact.org/project/qpaxQtz-9/bf272979. Download FIG S2, TIF file, 2.1 MB. Copyright © 2021 Di Gregorio et al. 2021 Di Gregorio et al. https://creativecommons.org/licenses/by/4.0/ This content is distributed under the terms of the Creative Commons Attribution 4.0 International license.

In Argentina, the ST30-MRSA-IV clone was traditionally characterized by a narrow range of resistance to antibiotics other than penicillin and oxacillin (13). The majority of isolates carried *mecA* on *SCCmec* type IV (*N* = 189, 99.5%) and *blaZ* genes (*N* = 185, 97.4%), conferring resistance to beta-lactams. We also identified independent acquisitions of other AMR determinants mostly within clade ARG-4, albeit in low frequency and without any signatures of resistance-related clonal expansions in the tree (see Fig. S2).

Of note, seven strains within ARG-4 harbored a plasmid with the *rep15* replicon associated with plasmid conjugation transfer (*tra*) genes and at least one AMR gene (*ileS2*, *lnuA*, *aacA*, *aacA-aph2″*, *ant4*, and/or *smr-qacC*) in addition to *mecA* and *blaZ*. This suggests that these strains might be carrying a conjugative plasmid conferring multiple antimicrobial resistance (27), a finding not yet reported for CC30-MRSA-IV in our country (Fig. S2).

The genotypic predictions of antibiotic resistance were compared against available phenotypic data, and the resulting overall concordance was 99.044%, with only between 1 and 4 discordant results found for erythromycin, clindamycin, linezolid, gentamicin, and ciprofloxacin (see Table S4).

10.1128/mSphere.01297-20.9 TABLE S4 Comparison of antibiotic resistances predicted *in silico* and phenotypic antimicrobial susceptibility test (AST) results. Download Table S4, XLSX file, 0.01 MB. Copyright © 2021 Di Gregorio et al. 2021 Di Gregorio et al. https://creativecommons.org/licenses/by/4.0/ This content is distributed under the terms of the Creative Commons Attribution 4.0 International license.

Interestingly, the genomes also provided information about other antimicrobials not routinely tested in the laboratory. Fosfomycin is not commonly used to treat staphylococcal infections in Argentina and was thus not included in the antibiotic panel. Surprisingly, all strains were predicted to be resistant to fosfomycin, as they carried the acquired *fosB* gene (*N* = 188) and mutations in the *murA* gene (D278E and E291D, *N* = 190).

### Virulence determinants of prevalent CC30 MRSA clades in Argentina.

Previous results that showed virulent behavior of MRSA-ST30-IV in animal models of infection (24) led us to characterize the virulence gene profile of this strain collection. Many virulence genes are notoriously associated with prophages or staphylococcal pathogenicity islands (SaPIs), and their distribution may vary between clones and even between closely related strains (28, 29). Most genomes carried prophages of the Sa2*int* type (*N* = 182) and *lukS/F-PV* genes (*N* = 181), but clade-specific differences were observed in the *lukS/F-PV* alleles (haplotype) and in the phage morphological group (Table 1; Fig. S3). Additionally, the distribution of prophages of the Sa3*int* and Sa1*int* types and of SaPI types (*int*SaPI2, *int*SaPI4, and *int*SaPI3-like) exhibited clade-specific differences (Table 1, Fig. S3, and Table S2).

10.1128/mSphere.01297-20.3 FIG S3 Distribution of virulence genes and associated MGEs. Midpoint-rooted phylogenetic tree as in Fig. 1 and colored by clade (ARG-1, turquoise; ARG-2, pink; ARG-3, light green; and ARG-4, dark blue). Colored blocks indicate the distribution of phage integrases (light blue), SaPI integrases (navy), PVL-encoding phage head type (characterized from assemblies as phiPVL phages with icosahedral or elongated heads), the PVL haplotype, and virulence genes (magenta). Absence of a genetic determinant is colored in pale grey. Data are available at https://microreact.org/project/qpaxQtz-9/f6534bf1. Download FIG S3, TIF file, 0.3 MB. Copyright © 2021 Di Gregorio et al. 2021 Di Gregorio et al. https://creativecommons.org/licenses/by/4.0/ This content is distributed under the terms of the Creative Commons Attribution 4.0 International license.

**FIG 2:**
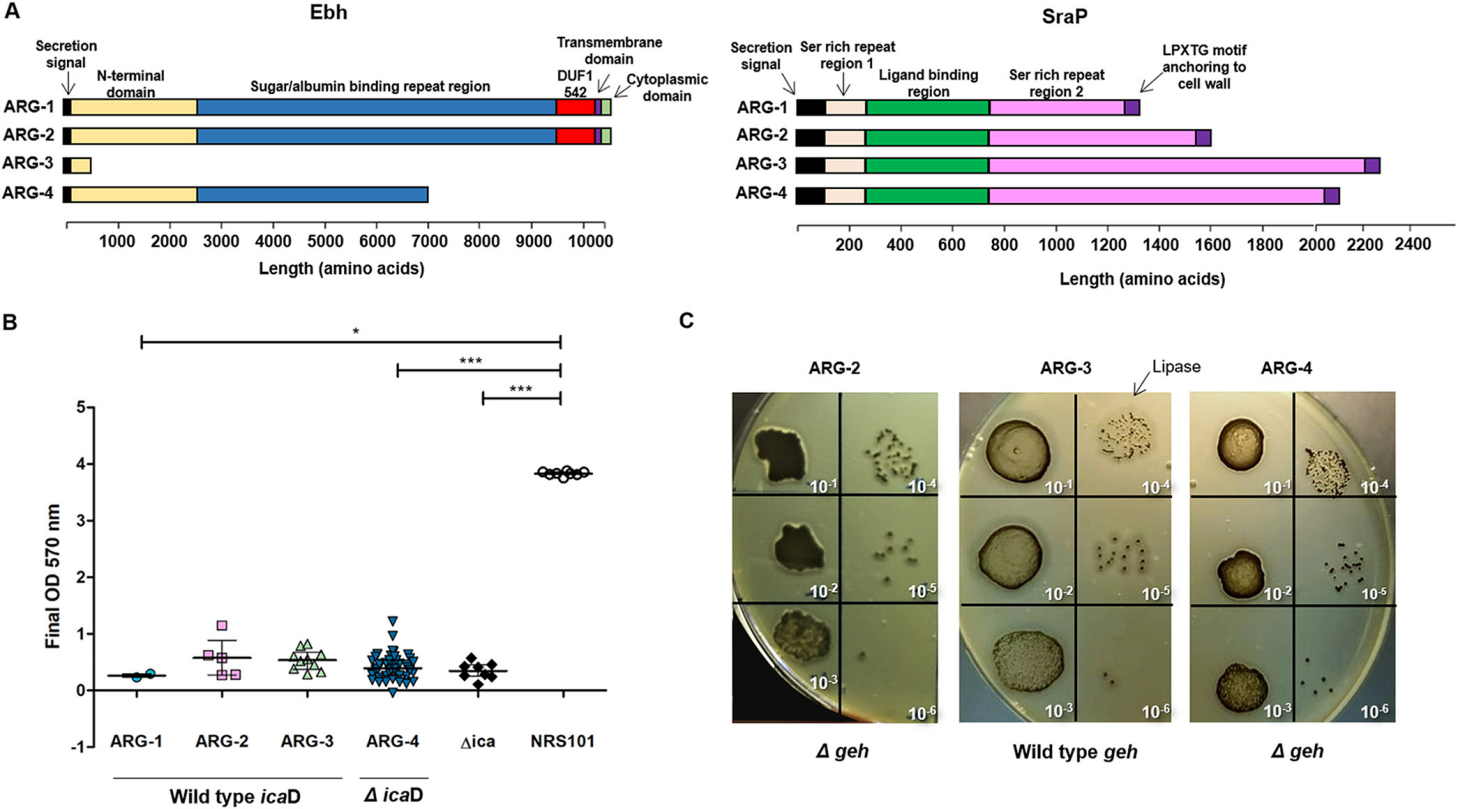
Virulence of Argentinean CC30 MRSA major clades. (A) Schematic representation of predicted Ebh and SraP protein sequences and domains in the four clades. (B) Biofilm production by isolates of ARG-1 (*N* = 2), ARG-2 (*N* = 5), ARG-3 (*N* = 10), and ARG-4 (*N *= 45). Results are represented as final OD for each strain with median value (line) and interquartile range (whiskers) and were compared with Kruskal-Wallis test (*P* < 0.0001). The horizontal bars show significant differences between individual groups detected by Dunn’s multiple-comparison test. *, *P* < 0.05; ***, *P* < 0.001. NRS101 (S. epidermidis NRS101), strong biofilm producer; Δ*ica* (S. aureus Newman Δ*ica*), *ica* independent-biofilm producer strain. (C) Lipase activity on Baird-Parker agar plates. For each representative isolate of ARG-2, ARG-3, and ARG-4, 10 μl of 10-fold serial dilutions of a 0.5 McFarland suspension were plated.

We also noticed clade-specific genetic variation in 4 genes (*icaD*, *geh*, *ebh*, and *sraP*) among the large repertoire of virulence genes found in all major clades (Table S2). The complete genes from the intercellular adhesion (*ica*) locus involved in adherence and biofilm formation (30) were present across all genomes (Table S2), with the exception of *icaD*. Strains within ARG-4 harbor an indel in *icaD* (delT255) causing a premature stop codon, thus resulting in a predicted protein 11 amino acids shorter than the reference sequence. Likewise, the genomes belonging to ARG-2 and ARG-4 harbored a SNP in *geh* (C106T) leading to a premature stop codon and resulting in a predicted protein of 35 amino acids corresponding only to the signal peptide domain of the Geh lipase (31). Moreover, the chromosomal virulence genes coding for the giant surface-bound proteins Ebh (extracellular matrix [ECM]-binding protein homologue) and SraP (serine-rich adhesin for platelets protein), involved in an ArlR-MgrA cascade controlling clumping and adhesion (32), presented clade-specific SNPs and indels in their repetitive regions, leading to proteins of different size. Remarkably, the genetic changes in Ebh found in ARG-3 and ARG-4 genomes lead to predicted truncated proteins that lacked the transmembrane region and therefore should not be anchored to the membrane (Fig. 2A).

To evaluate the phenotypic significance of the observed genetic changes, representative strains from the 4 major clades were assayed for biofilm formation and lipase activity. All CC30 strains analyzed displayed low biofilm production. Surprisingly, the *icaD* mutation harbored exclusively by strains within ARG-4 did not seem to further impact biofilm formation, as there was no significant difference with other clades (*P* > 0.05, Kruskal-Wallis with Dunn’s *a posteriori* test) (Fig. 2B). In contrast, representative isolates of ARG-2 and ARG-4 carrying a truncated version of Geh showed a reduction in lipase activity compared to that in strains belonging to ARG-3 when tested on Baird-Parker agar plates (Fig. 2C).

### Genetic diversification of clade ARG-4.

In addition to differences in the accessory genome, the clonal expansion of clade ARG-4 could be linked to the acquisition of adaptative mutations. We defined the core genetic changes within this clade using the phylogeny in Fig. 1 as a framework, with a focus on those SNPs that might help explain the success of ARG-4. We found 440 core SNPs shared between all the genomes within this clade (*N* = 164), of which, 221 resulted in nonsynonymous mutations (see Table S5). Among them, the most relevant changes included mutations in the transcriptional regulators *arlR* (A14V) and *sarX* (G253A) and 6 SNPs leading to premature stop codons in genes ILRI_01209, *tag*, *rplV*, *hsaA*, *yghA*, and *cocE* (Table 2). The expression of dehydrogenase genes *hsaA* and *yghA* and of *cocE* was previously found to be affected by long-chain unsaturated free fatty acids (LCuFFAs), while the *rplV* gene coding for 50S ribosomal protein L22 is implicated in protein synthesis.

**TABLE 2:** SNPs leading to premature STOP codons in clades ARG-3 and ARG-4^*a*^

Position	Gene name	Product	SNP
ARG-4 (*N* = 164)			
1280667	*ILRI_01209*	Hypothetical protein	G94A
1747625	*tag*	DNA-3-methyladenine glycosylase	T149A
2369884	*rplV* ^*b*^	50S ribosomal protein L22	T343A
2406078	*hsaA* ^*c*^	Acyl-CoA dehydrogenase	C55A
2448271	*yghA* ^*c*^	Short-chain dehydrogenase	C250A
2730310	*cocE* ^*c*^	X-pro dipeptydil peptidase (S15) family protein	C715T
309872	*geh*	Glycerol ester hydrolase	C106T
ARG-3 (*N* = 19)			
105294	*phnC*	Phosphate import ATP binding protein	G460A
1283886	*ILRI_01212*	Hypothetical protein	G73T
1635067	*ILRI_01607*	Competence ComGF-like protein	G352A
2053078	*map_3*	MHC class II antigen-like protein	G1603A
2085979	*mutS_2*	MutS-related protein	G237A
2508488	*ydaG* ^*c*^	General stress protein 26	C271T

a Only SNPs present in whole genomes after removing MGEs and recombination regions are shown. Positions are in reference to the ST30 S. aureus ILRI_Eymole1/1 reference genome. Genes without an annotated name are in reference to CDS of the same reference genome by their locus tag.

b Genes involved in protein synthesis.

c Genes described to be affected by LCuFFAs.

10.1128/mSphere.01297-20.10 TABLE S5 Core SNPs found in clade ARG-4. Only SNPs present in whole genomes after removing MGE and recombination regions are shown. Positions are in reference to the ST30 S. aureus ILRI_Eymole1/1 reference genome. Genes without an annotated name are in reference to the CDS of the same reference genome by their locus tag. Download Table S5, XLSX file, 0.1 MB. Copyright © 2021 Di Gregorio et al. 2021 Di Gregorio et al. https://creativecommons.org/licenses/by/4.0/ This content is distributed under the terms of the Creative Commons Attribution 4.0 International license.

**FIG 3:**
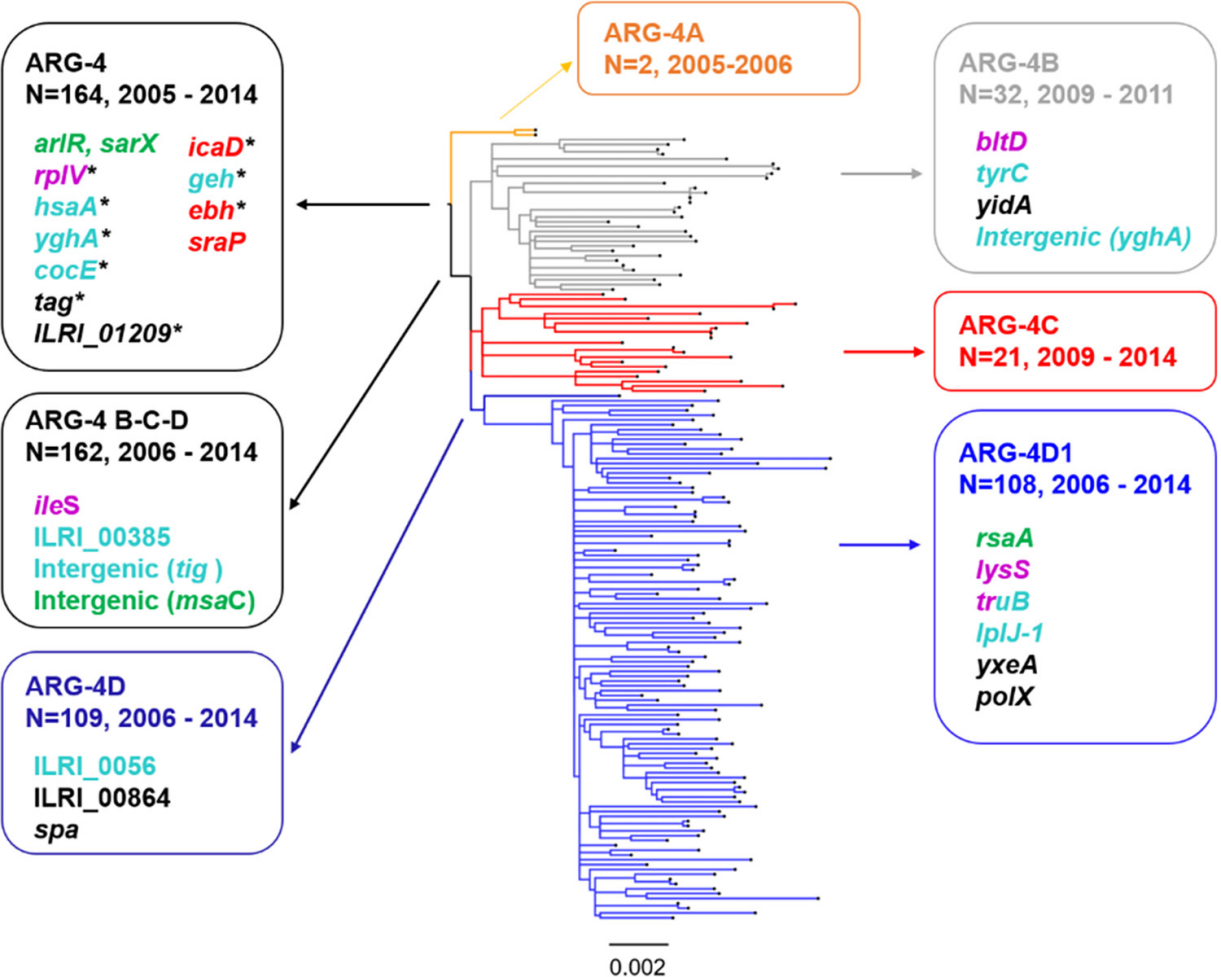
Distribution of selected mutations in the phylogeny of clade ARG-4. Midpoint-rooted phylogenetic tree inferred as described for Fig. 1. Subclades within ARG-4 are colored in orange (ARG-4A), gray (ARG-4B), red (ARG-4C), and blue (ARG-4D). The scale bar represents the number of single nucleotide polymorphisms (SNPs) per variable site. The SNPs unique to each labeled subclade or internal node (arrows) are annotated within the boxes. Genes without an annotated name are in reference to the coding DNA sequence (CDS) of ILRI_Eymole1/1 reference genome by their locus tag. Gene names are colored by function as follows: protein synthesis (purple), response to free fatty acids and lipase *geh* (aqua), clumping/adhesion/biofilm (red), transcriptional regulators (green). SNPs leading to a premature stop codon are annotated with an asterisk.

The tree topology of clade ARG-4 showed early diversification into four subclades with bootstrap values higher than 83% (ARG-4A, -4B, -4C, and -4D) (Fig. 3). While the geographic distribution of clades ARG-4A and ARG-4C was circumscribed to Buenos Aires, ARG-4B and ARG-4D were found in all provinces included in this study, with ARG-4D (*N* = 109) showing the largest clonal expansion. Remarkably, we found additional SNPs in genes involved in protein synthesis and the response to free fatty acids, both in the branch leading to subclades ARG-4B-C-D (*N* = 162) and within each of the individual subclades (Table 3; Fig. 3). Moreover, genomes within subclade ARG-4D1 (*N* = 108) harbored a nonsynonymous mutation located in the H1 loop of *rsaA* (T45A), a noncoding RNA with regulatory functions and linked to virulence (33). The *rsaA* RNA activates biofilm formation and inhibits capsule synthesis by repressing the global transcriptional regulator MgrA through interaction with the mRNA via the H1 and H2 loops (33). Nevertheless, we found no significant differences in biofilm formation between representative isolates of subclade ARG-4D1 and isolates from the other subclades within ARG-4 (see Fig. S4).

**TABLE 3:** SNPs defining subclades within clade ARG-4^*a*^

Position	Gene	Product	Effect
Subclades ARG4B-D (*N* = 162)			
1249301	*ileS* ^*b*^	Isoleucyl tRNA synthetase	Nonsynonymous
407511	*ILRI_00385* ^*c*^	Lipoprotein	Nonsynonymous
1759464	Intergenic (109 bp upstream *tig*^*c*^)		
1005326	Intergenic (44 bp upstream *msaC*)		
Subclade ARG-4B (*N* = 32)			
865696	*yidA*	Hydrolase, sugar phosphate phosphatase	Nonsynonymous
1042381	*tyrC* ^*c*^	Arogenate dehydrogenase, prephenate dehydrogenase	Nonsynonymous
2447638	Intergenic (1 bp downstream *yghA*^*c*^)		
487644	*bltD* ^*b*^	Acetyltransferase, spermine/spermidine acetyltransferase	Nonsynonymous
2827657	Intergenic (215 bp downstream *lipA_3*)		
Subclade ARG-4C (*N* = 21)			
891779	Intergenic (120 bp upstream *ILRI_00864*)		
2446074	*metT*	Methionine transporter	Synonymous
1657754	*glyS* ^*b*^	Glycyl-tRNA synthetase	Synonymous
Subclade ARG-4D (*N* = 109)			
67210	*ILRI_0056* ^*c*^	Myosine cross-reactant/oleate hydratase	Nonsynonymous
1187549	Intergenic (32 bp upstream *ILRI_01121*)		
891579	Intergenic (upstream *ILRI_00864*)		
891680	Intergenic (upstream *ILRI_00864*)		
891754	Intergenic (upstream *ILRI_00864*)		
891906	*ILRI_00864*	Lipoprotein	Nonsynonymous
71338	*spa*	Staphylococcal protein A	Nonsynonymous
Subclade ARG-4D1 (*N* = 108)			
2510226	*gltT* ^*c*^	Proton glutamate symport protein	Synonymous
2070776	Intergenic (43 bp upstream *amiE*)		
2508340	*ydaG* ^*c*^	General stress protein 26	Synonymous
590301	*rsaA*	Noncoding RNA	
1461525	*lplJ-1* ^*c*^	Lipoate-protein ligase A	Nonsynonymous
2113311	*lysS* ^*b*^	Lysyl-tRNA synthetase	Nonsynonymous
1156444	*truB* ^*b*^ ^,^ ^*c*^	tRNA pseudouridine synthetase B	Nonsynonymous
2060923	*ktrB-2*	Na^+^-transporting ATP synthase	Synonymous
406909	*yxeA*	Hypothetical protein	Nonsynonymous
1299928	*polX*	DNA polymerase X family	Nonsynonymous

a Only SNPs present in whole genomes after removing MGE and recombination regions are shown. Positions are in reference to the ST30 S. aureus ILRI_Eymole1/1 reference genome. Genes without an annotated name are in reference to the CDS of the same reference genome by their locus tag.

b Genes involved in protein synthesis.

c Genes described to be affected by LCuFFAs.

10.1128/mSphere.01297-20.4 FIG S4 Biofilm production by major subclades within ARG-4. Number of isolates tested by subclade: ARG-4B, *N* = 13; ARG-4C, *N* = 16; ARG-4D1, *N* = 16. Results are represented as the final OD for each strain with median value (horizontal line) and interquartile range (whiskers) and were compared with the Kruskal-Wallis test (*P* = 0.0008). The horizontal bars show significant differences between individual groups detected by Dunn’s multiple-comparison test. **, *P* < 0.01; ***, *P* < 0.001. NRS101 (S. epidermidis NRS101), strong biofilm producer; Δ*ica* (S. aureus Newman Δ*ica*), *ica*-independent biofilm producer strain. Download FIG S4, TIF file, 0.3 MB. Copyright © 2021 Di Gregorio et al. 2021 Di Gregorio et al. https://creativecommons.org/licenses/by/4.0/ This content is distributed under the terms of the Creative Commons Attribution 4.0 International license.

### Argentinean CC30 MRSA in a global context.

**FIG 4:**
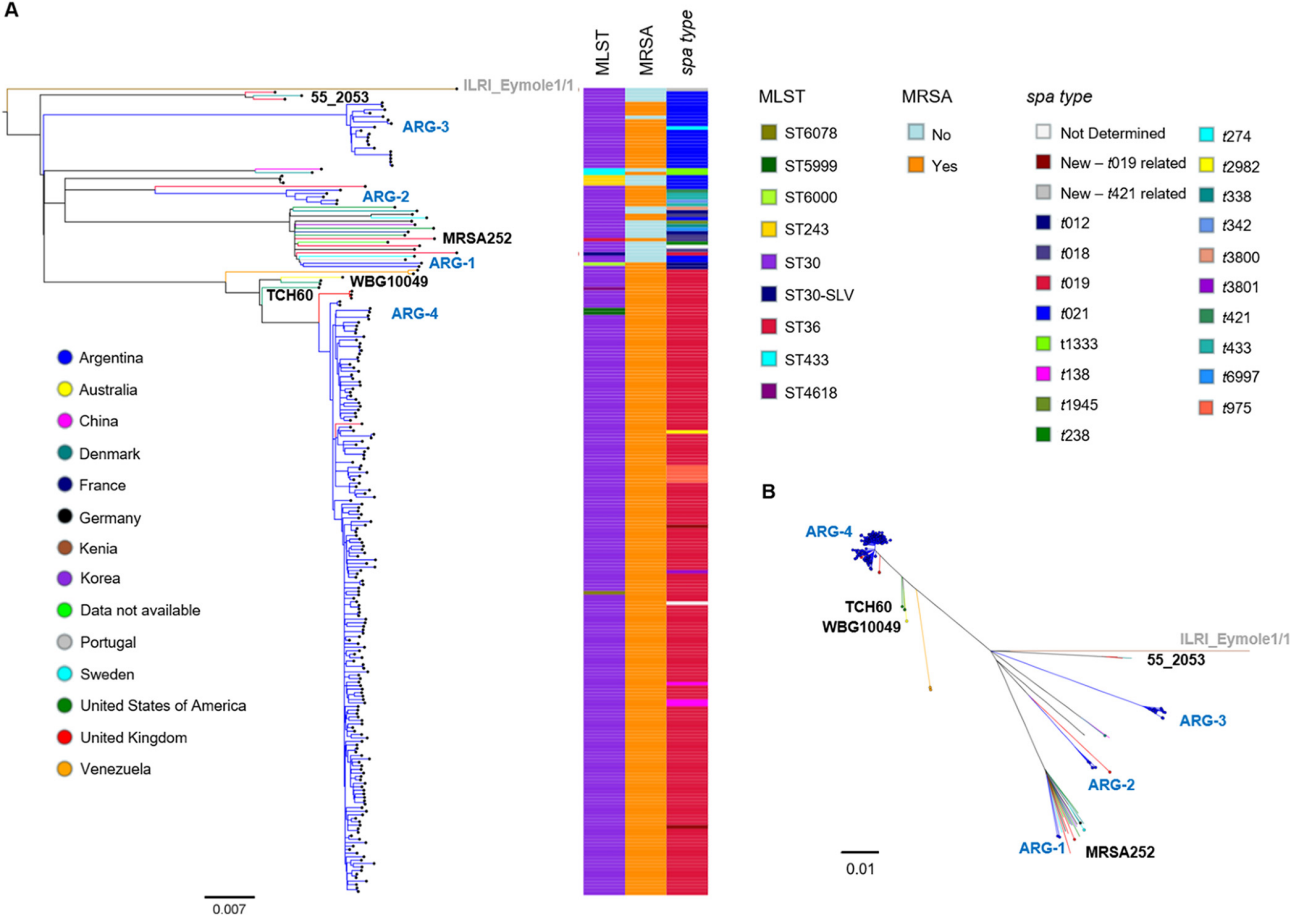
Argentinean CC30 MRSA strains in a global context. (A) Midpoint-rooted phylogenetic tree from 11,656 SNPs obtained after mapping the genomes to the complete genome of strain ILRI_Eymole1/1 (gray) and masking regions of recombination and MGEs. Tree branches are colored by country of origin as described in the legend. The distribution of genotypes is represented as metadata blocks and colored as described in the figure. Representative reference genomes of previously described CC30 lineages are labeled in black, and Argentinean major clades are represented labeled in light blue. (B) A radial (unrooted) version of the tree in panel A; MRSA strains are highlighted with circled nodes in this tree. Scale bars represent the number of single nucleotide polymorphisms (SNPs) per variable site. The data are available at https://microreact.org/project/SyLHdx6-E/aef7dbcd.

To provide a broader context to the Argentinean genomes, we reconstructed the phylogeny of the genomes in this study combined with 41 publicly available CC30 genome sequences from 14 countries, including representatives of the three successful CC30 epidemic lineages: phage type 80/81 (55_2053, ST30-MSSA), SWP (WBG10049; ST30-MRSA-IV), and EMRSA-16 (MRSA252; ST36-MRSA-II) (Fig. 4).

The Argentinean genomes did not form a monophyletic group (Fig. 4). The two Argentinean strains recovered from healthy children (ARG-1) clustered within a group of 18 genomes (100% bootstrap support) from diverse locations (United Kingdom, Europe, United States, and South Korea) including that of MRSA252 (EMRSA-16) (34). The closest relative to the five genomes in ARG-2 was contemporary strain BSAC698 isolated in England (35). Remarkably, none of the public genomes included in this phylogeny clustered with ARG-3, which forms a separate clade on a long branch (100% bootstrap support). On the other hand, ARG-4 is related (100% bootstrap support) but can be clearly distinguished from the less contemporary genomes belonging to the SWP clone of Australia (WBG10049, average 266 ± 9 SNPs) and related strains from North America (NRS484, USA1100, and TCH60). The only five previously described genomes from Argentina isolated between 2011 and 2014 (26) and one genome from the United Kingdom (3) were interspersed with the ARG-4 genomes from this study.

We further investigated whether the MGEs carried by ARG-4 were also present in the SWP strains and whether they had diversified. The *rep21* plasmid was conserved with the pTCH60 and pWBG10049 plasmids from SWP strains (>99% sequence identity). Likewise, the SaPI (*int*SaPI3-like) harbored by clade ARG-4 was conserved with those found on strains from the SWP lineage as well as on USA500 strain MN-1310 (36) (>99% sequence identity) (see Fig. S5). Surprisingly, the PVL-encoding phage type differed between clade ARG-4 and SWP strains (Tables S2 and S3). Despite having the same *lukSF-PVL* haplotype (H2a), the majority of clade ARG-4 harbor PVL phages with icosahedral heads (phiPVL-CN125-like; NC_012784), whereas older genomes of the SWP lineage harbor PVL phages with elongated heads (phiPVL-TCH60-like).

10.1128/mSphere.01297-20.5 FIG S5 Comparison of *int*SaPI3-like pathogenicity islands visualized with ACT. The red vertical blocks indicate conserved regions detected by pairwise BLASTN comparisons. SaPI Tokio 11212 (accession AB860416) is a pathogenicity island from staphylococcal food poisoning isolates. UBA71 (accession ERR3290891) is a representative strain from clade ARG-4. TCH60 (accession SRR014925) and NRS484 (accession SRR1955626) are two strains belonging to CC30 SWP lineage. MN1310 (accession SRR3418435) is a strain belonging to CC8 lineage. Download FIG S5, TIF file, 0.8 MB. Copyright © 2021 Di Gregorio et al. 2021 Di Gregorio et al. https://creativecommons.org/licenses/by/4.0/ This content is distributed under the terms of the Creative Commons Attribution 4.0 International license.

10.1128/mSphere.01297-20.8 TABLE S3 Characteristics of CC30 S. aureus global genomes included in the analysis. “yes,” presence of a genetic determinant; “no,” absence of a genetic determinant; ND, not determined; M, male; F, female; SLV, single locus variant; SSSI, skin and skin structure infection. Download Table S3, XLSX file, 0.1 MB. Copyright © 2021 Di Gregorio et al. 2021 Di Gregorio et al. https://creativecommons.org/licenses/by/4.0/ This content is distributed under the terms of the Creative Commons Attribution 4.0 International license.

## DISCUSSION

Clonal complex 30 (CC30) groups pandemic Staphylococcus aureus lineages that have been consistently reported from all continents (8, 37), including ST30-MRSA-IV, one of the most prevalent MRSA lineages in Argentina (13, 17). This is the first extensive genomic study of CC30 MRSA in Argentina, describing the genetic diversity, population structure, and genomic characteristics of strains collected between 2004 and 2015.

The CC30 population was composed of 4 major clades with different repertoires of AMR and virulence determinants and MGEs: ARG-1 (CC30-IVc-*spa* t012), ARG-2 (ST30-IVc *spa* t021 and related), ARG-3 (ST30-IVh/j-*spa* t021 and related), and ARG-4 (CC30-IVc-*spa* t019 and related) (Fig. 1 and 2; Table 1). Previous surveillance studies documented the presence of ST30-MRSA-IV-*spa* t019 as a prevalent clone in the country and detected ST30-MRSA-IV with *spa* t021 and/or t012 as minority clones (12, 13, 17).

The same studies also reported low rates of resistance to non-beta-lactam antibiotics among ST30-MRSA-IV strains (12, 13, 17). Our results confirmed those findings and additionally revealed the resistance mechanisms and MGEs responsible for this sporadic acquisition of resistance to non-beta-lactam antibiotics (see Fig. S2 in the supplemental material). Moreover, the AMR phenotype-genotype concordance for the antibiotics tested was high, in agreement with genomic studies from other countries (25, 38), thus showing that the strain collection studied here does not present local differences in terms of uncharacterized AMR mechanisms. Surprisingly, the availability of whole-genome sequences uncovered genetic determinants of antimicrobial resistance to fusidic acid (*fusA* H457Y and L461F), mupirocin (*ileS-2*), fosfomycin (*murA* D278E and E291D and *fosB*), and quaternary ammonium compounds (*smr-qacC*), some of them described for the first time in Argentinean isolates (Fig. S2). Of concern, the presence of plasmids carrying *smr-qacC* in 19 strains within clade ARG-4 from 10 different hospitals might be the result of selective pressure exerted by antiseptic use as infection prevention and/or exposure to sublethal concentrations of disinfectants as a consequence of poor disinfection practices in health care settings (39).

The population was dominated by clone ARG-4 (Fig. 1), a successful lineage phylogenetically related to the SWP clone (Fig. 4), which has persisted and spread throughout the country (12, 13, 17, 24). The phylogeny and accessory genome showed that ARG-4 was highly clonal, albeit without phylogeographic structure (Fig. 1). This observation may suggest a lack of locally adapted lineages of ARG-4, especially in Buenos Aires and Misiones, although we cannot rule out that the lack of phylogeographic clustering may be due to the different sampling strategies of the strains studied here.

We had previously characterized the virulence of ST30-MRSA-IVc-*spa* t019 with molecular methods and animal models of infection (24). Using WGS, we identified clade-specific genetic changes in genes linked to virulence and fitness, in particular, in clade ARG-4. These observations, together with the clonal expansion and success of this clade in Argentina, prompted us to speculate that they could be of adaptative value (17, 24). Geh (glycerol ester hydrolase) is a lipase with a preference for long-chain fatty acid esters (such as those found on skin or in egg yolk lecithin) and has a role in both S. aureus colonization and infection (31, 40). The observed reduction in lipase activity (Fig. 2C) aligned with the truncation of *geh*, together with the cumulative changes observed in genes related both to the response to free fatty acids present on skin and to protein synthesis (41, 42) (Fig. 3 and Tables 2 and 3), opens the possibility that clade ARG-4 strains may have undergone an adaptation related to skin colonization.

Also, the combined effects of changes in virulence-related genes may hint to differences in adhesion to endothelial cells, clumping, and dissemination of clade ARG-4 strains. The noncoding RNA *rsaA* and the *arlRS* operon act on the global transcriptional regulator MgrA (32, 33), which in turn represses the expression of giant surface proteins Ebh, SraP, and SasG (inhibiting adhesion and clumping in a dose-, location-, and size-dependent manner) and activates virulence regulator SarX (32, 43). Although none of the 190 genomes carried mutations in *mgrA* or the *sasG* gene, we found cumulative changes within the ARG-4 clade in genes along the regulatory cascade described above (*sarX*, *arlR*, *rsaA*, *ebh*, and *sraP*) and in other virulence-associated genes (*icaD*, *geh*, and *ileS*) (Fig. 3) (44). Nevertheless, other genetic changes playing a role on S. aureus evolution, such as indels or the transposition of insertion sequences (36, 45), should not be disregarded, as they could also have been involved in the selection of ARG-4 and the adaptation of the sublineages.

Whether any of these genetic changes, alone or in combination, is responsible for the reported ability of ST30-MRSA-IV-*spa* t019 to disseminate into body tissues (24) and for its low adherence to plastic surfaces (biofilm formation) (Fig. 2) remains to be studied, as a characterization of the ARG-4 virulence phenotype was beyond the scope of this study.

Several genetic mechanisms regulate biofilm formation in staphylococci, acting on its expression and composition (46). Hence, the observed biofilm phenotype of CC30 strains may not necessarily reflect the impact of mutations in the *icaADBC* operon, as the expression of this locus has been found to depend on the environmental conditions and the strain genetic background (47). Likewise, the consistently low biofilm formation observed among CC30 clades, regardless of the presence of intact or truncated *icaD* (Fig. 2; Fig. S4), could also be due to unexplored additional genetic changes in *ica*-dependent and *ica*-independent pathways that govern biofilm formation (such as the lack of the *sasG* gene in all strains and/or changes in the activity of the *agr* locus, *SpA*, FnbPs, etc.) (46). Biofilm formation in CC30 strains differed from that in our previous results (24) (Fig. 2). This might be, at least in part, explained by the higher number and different set of strains used and the different conditions assayed in this study.

Previous epidemiological studies used information of MLST, *spa*, and PFGE types to link Argentinean ST30-MRSA-IV-pulsotype C-*spa* t019 with the SWP clone described in Australia (12, 17). Interestingly, phylogenetic analysis performed here revealed that ARG-4 circulating in Argentina would be a distinct lineage from that evolved from the SWP clone since its first description in 1999 and is also present in the United Kingdom (Fig. 4) (3, 48). A similar observation was recently reported by a surveillance study of S. aureus in the Philippines (49), which described the recent diversification of the local CC30-MRSA-IV-*spa* t019 from the SWP clone and their close relationship to public global genomes, including previous genomes from Argentina included in Fig. 4. Relevant genetic features also distinguished ARG-4 from other genomes of the SWP lineage, such as the phiPVL phage type (Tables S2 and S3) and mutations in *ebh*, *sraP* (coding for surface proteins), and *tag* (DNA-3-methyladenine glycosylase) (Table 2). Previous studies analyzing the CC30 lineage found that SWP strains were more likely to have PVL-encoding phages with elongated heads (8, 50), but clade ARG-4 harbored those with icosahedral heads despite having the same *lukS/F-PVL* genes. More studies are necessary to determine if icosahedral-headed PVL phages found in ARG-4 (phiPVL-CN125-like) are prevalent within SWP lineage elsewhere and whether these different prophages result in biological differences affecting the expression of the PVL and the virulence of these two clades. Furthermore, genetic differences in genes containing large variable repeats, such as *ebh* and *sraP*, were described to be lineage associated (51, 52), reflecting the evolution of the ARG-4 clade through the accumulation of changes in those genes.

In addition to the genetic characterization of ARG-4, our findings provide evidence of a community reservoir of successful international CC30 MRSA lineages other than SWP in Argentina, such as ARG-1 and ARG-2 with links to strains circulating globally (Table 1; Fig. 4) (25, 35, 53). Of note, ARG-1 also carries *tst-1* on a SaPI2-like structure and mutations in AgrC (G55R) and Hla (premature stop), both characteristic of the EMRSA-16 lineage (8, 9).

Furthermore, our work contributes to the global knowledge of the CC30 population structure. For example, clade ARG-3 showing a geographic distribution mainly limited to the city of Posadas shares some characteristics with the phage 80/81 lineage (ST30, *spa* t021, PVL^+^) but does not cluster with any of the representative global strains included in our analysis (Fig. 4) and might constitute, therefore, a novel lineage within CC30. Relevant genetic markers of this new lineage include accessory genes and MGEs listed in Table 1 and 6 mutations leading to premature stop codons (Table 2). Due to the composition of our strain collection, the geographic and host distribution of this new lineage remains to be explored.

We recognize that all samples analyzed herein derived from previous surveillance studies that followed different sampling strategies; thus, the representation of locations, time points, and infection types (SSSI/invasive and/or acute/chronic) is uneven. In addition, isolates may differ in the expression of certain virulence factors during chronic infections (54), even though this does not affect the phylogenies generated here. Our collection represents only a small number of Argentinean provinces, but it includes provinces with higher prevalence of MRSA (12). Our study represents the first genome-based description of CC30-MRSA in Argentina and provides an in-depth characterization of the genetic AMR and virulence mechanisms of a successful S. aureus lineage in our country. The genomes generated will serve as baseline genomic data going forward with the implementation of S. aureus genomic surveillance in the region. Expanding WGS-based surveillance to more hospitals and provinces of Argentina is necessary to completely understand the epidemiology and dynamics of S. aureus lineages in our country. This will allow to address the development of new diagnostic and typing methodologies to detect high-risk clones and to improve infection control.

## MATERIALS AND METHODS

### Bacterial isolates.

All isolates previously characterized in our laboratory as MRSA by their resistance to oxacillin-cefoxitin and as CC30 by MLST were included and sequenced in this study (*N* = 190) (summarized in Table S1 in the supplemental material). These derived from different surveillance studies analyzing invasive infections, skin and skin structure infections (SSSIs), patients with cystic fibrosis (CF), or healthy carriers in Argentina (13, 16–20, 55, 56) and were recovered between 2004 and 2015 from 26 hospitals and 1 kindergarten located in three Argentinean provinces (Buenos Aires, Santa Fé, and Misiones). Epidemiological metadata (age and gender of patients, hospital, infection type, and date) and information on antimicrobial susceptibility and pulsed-field gel electrophoresis (PFGE) pulsotypes as described earlier (17) can be found in Table S2 and in the Microreact project available at https://microreact.org/project/qpaxQtz-9.

All isolates were previously tested for antimicrobial susceptibility to different antibiotics according to Clinical and Laboratory Standards Institute (CLSI) guidelines (57, 58). Discordances between phenotypic antibiotic susceptibility profiles and WGS results from this study were confirmed using disk diffusion tests according to CLSI guidelines (57).

### Whole-genome sequencing, assembly, and annotation.

Genomic DNA was extracted using the QIAcube HT system (Qiagen) with the addition of lysostaphin. DNA was quantified with the Qubit 3.0 fluorometer (Invitrogen). Whole-genome sequencing was performed on the Illumina HiSeq X 10 platform with paired-end reads (2 by 150 bp). Annotated assemblies were produced as previously described (59). Briefly, for each sample, sequence reads were used to create multiple assemblies using VelvetOptimiser v2.2.5 (60) and Velvet v1.2 (61). Contigs were scaffolded using SSPACE v2.0 (62), and sequence gaps were filled using GapFiller v1.11 (63). Automated annotation was performed using PROKKA v1.5 (64) and a genus-specific database from RefSeq (65). Quality control of sequence data was performed using the Wellcome Sanger Institute Pathogen Informatics QC pipeline based on (i) the basic stats of raw reads, (ii) the assembly stats, (iii) the mapping stats generated by randomly sampling 100 Mb from each sample and aligning it to reference genome of S. aureus strain TW20 (accession FN433596.1 [66]), (iv) the number of heterozygous SNPs (based on the 100 Mb alignment), and (v) the proportion of reads of each sample assigned to each taxon in the RefSeq database with Kraken v1.1 (67) (Table S2). A given position in the genome is considered heterozygous if (i) the total read depth on each strand is ≥4, (ii) it has at least 2 variants where number of reads supporting variant is ≥2, and (iii) (number of reads supporting variant)/(total depth) ≤ 0.9.

### Variant detection and phylogenetic analysis.

Paired-end reads were mapped against the S. aureus ILRI_Eymole1/1 reference genome (ST30, accession NZ_LN626917) (68) using Burrows-Wheeler alignment (BWA) v0.7.17 (69). Variants were called with SAMtools mpileup v0.1.19 (70) with parameters -d 1000 -DSugBf and bcftools v0.1.19 (71) to produce a BCF file of all variant sites. A pseudogenome was constructed by substituting the base call at each site (variant and nonvariant) in the BCF file into the reference genome, and any site called as uncertain was substituted with an N. Insertions with respect to the reference genome were ignored, and deletions with respect to the reference genome were filled with Ns in the pseudogenome to keep it aligned and the same length as the reference genome used for read mapping. A whole-genome alignment was created after masking known MGE regions and variable sites associated with recombination (detected with Gubbins v2.3.2 [72]). The resulting alignment of polymorphic sites was then used to construct a maximum likelihood (ML) phylogenetic tree using RAxML version 8.2.8 (73), based on the generalized time reversible (GTR) model with GAMMA method of correction for among-site rate variation and 100 bootstrap replicates. SNPs were reconstructed against the tree and pairwise SNP differences were calculated with https://github.com/sanger-pathogens/bact-gen-scripts/blob/master/reconstruct_snps_on_tree.py and https://github.com/sanger-pathogens/bact-gen-scripts/blob/master/pairwise_difference_count.py, respectively.

To provide a broader geographic context to the data, we performed a second phylogenetic analysis following the method described above but also including 41 publicly available S. aureus genomes belonging to CC30. Global genomes were selected to include representatives of the 3 major CC30 pandemic lineages (8–10) and the only seven published CC30 genomes from Latin America (26). The 41 global genomes included in this tree and their accession numbers are listed in Table S3 and described in the Microreact project available at https://microreact.org/project/SyLHdx6-E/aef7dbcd.

### *In silico* genotyping.

The *spa* and MLST types were derived from assemblies using free online resources spaTyper (http://spatyper.fortinbras.us) and Pathogenwatch (https://pathogen.watch/), respectively. SCC*mec* IV subtyping was determined from reads using ARIBA v2.12.1 and a database of subtype IV-specific genes previously described (6, 74–78).

### Pangenome analysis.

The pangenome of the 190 Argentinean isolates was determined with Roary v3.12.0, using a blastp percentage identity of 95% and a core definition of 99% (79). SNPs in the core genome inferred with Roary v3.12.0 were identified with snp-sites v2.4.0 (80), and an ML phylogenetic tree was built with RAxML v8.2.8 (73). The distribution of accessory genes and their relationship with different phylogenetic groups were identified and visualized with Phandango (81).

### Detection of antibiotic resistance determinants, virulence genes, and mobile genetic elements.

Detection of antimicrobial resistance determinants, virulence genes, and MGEs was carried out with ARIBA v2.12.1 (78) and relevant databases. For antimicrobial resistance determinants, we used Resfinder (82), CARD (83), ARGANNOT (84), and a curated database (85). The BlaZ amino acid sequences derived from the ARIBA output were aligned, and positions 128 and 216 were compared to identify the BlaZ type as previously described (86). Virulence genes were detected using a database of 106 staphylococcal virulence genes (25). The *icaD*, *geh*, and *lukS/F-PV* nucleotide sequences assembled by ARIBA were aligned with MUSCLE on Seaview v4.7 and compared with reference sequences available from the virulence database. The nucleotide sequences of virulence genes that failed to assemble fully with ARIBA (*coa*, *essC*, *sspA*, *clfA*, *clfB*, *cna*, *fnbA*, *fnbB*, *sdrC*, *sdrD*, *bbp*, *sraP*, and *ebh*) were obtained by querying the genome assemblies with BLAST and a database of the gene sequences from CC30 reference genomes MRSA252 (NC_002952.2) and TCH60 (NC_017342.1). Plasmids types were defined based on their replicon genes (*rep*) using the Plasmidfinder database (87). Phage types were defined based on their integrase gene, using the 12 integrase groups described by Goerke et al. (88). Thirteen known staphylococcal pathogenicity islands (SaPIs) were queried based on their integrase (*int*) genes described by Subedi el al. (89). The co-occurrence on the same assembly contig of select combinations of AMR genes and plasmidic *rep* genes, or virulence genes and *int* genes, was verified on at least 2 representative genomes from each clade/subclade.

Integrative conjugative elements (ICEs) and phiPVL prophages were detected by querying the genome assemblies with BLASTN v2.7.1 and a custom database. ICEs were detected based on full-length sequences of ICE6013 (ICE6013 from strain MRSA252 [accession NC_002952.2] [34]), Tn*916* from Enterococcus faecalis strain DS16 (accession GCA_000147495.1), and Tn*5801* from S. aureus strain Mu50 (accession NC_002758 [90, 91]); PVL-encoding phages were characterized as phiPVL phages with icosahedral or elongated heads as per the reference sequences of the 7 phages known to carry the PVL genes (92, 93) and phiPVL-TCH60 (accession NC_017342.1). Different *lukSF-PV* alleles were detected according to the haplotype group characterization described by Chen et al. (50).

All genomes, MGEs, and genome comparisons with reference sequences of interest were visualized in Artemis and/or ACT (94, 95). The Microreact web application was used for the integrated visualization of phylogenetic trees, geographic and temporal data, and other associated epidemiological and genetic data (96) (https://microreact.org/project/qpaxQtz-9). The antimicrobial resistance determinants, virulence factors, and MGEs are provided in full in the Microreact project and in Table S2.

### Biofilm assay.

Biofilm development of 62 isolates (2 isolates from clade ARG-1, 5 from ARG-2, 10 chosen randomly from ARG-3, and 45 chosen randomly from ARG-4) was assessed by measuring the accumulation of biomass on the surface of sterile 96-well flat-bottom polystyrene plates (Extragene) using a method adapted from Stepanovic et al. (97). Briefly, 200 μl of a 1/100 dilution of a bacterial suspension adjusted to an optical density at 620 nm (OD_620_) of 0.2 (≈10^8^ CFU/ml) in tryptic soy broth (TSB) supplemented with sterile 1% glucose was added to wells (6 replicates per strain). Following 24 h of incubation at 37°C, the plate was washed twice with 0.9% NaCl and air dried for 2 h. The remaining attached bacteria were fixed for 15 min with 200 μl of methanol 99% (vol/vol) per well, after which, the plates were emptied and air dried. The plates were then stained for 20 min with 200 μl of 0.5% crystal violet per well. Finally, wells were washed with water and air dried, the dye was solubilized with 33% acetic acid solution, and the OD_570_ for each well was measured. S. aureus Newman Δ*ica* (non *ica*-dependent biofilm producer) and Staphylococcus epidermidis NRS101 (prototype biofilm producer) were included in the assay as control strains. Biofilm production was calculated as: final OD_570_ of test strain = average OD_570_ of test strain − ODc, where average OD_570_ is the average value of the six replicates, and ODc is the average OD_570_ value from the six replicates for the negative control (uninoculated broth) plus 3 standard deviations of the negative control.

### Lipase activity on Baird-Parker agar.

Tenfold dilutions of 0.5 McFarland suspensions (≈10^8^ CFU/ml) of 27 representative strains (2 from clade ARG-1, 5 from ARG-2, 10 chosen randomly from ARG-3, and 10 chosen randomly from ARG-4) were inoculated onto Baird-Parker agar (Britania) and cultivated for 24 h at 37°C. The presence of egg yolk in this medium permits the detection of lipolytic activity of staphylococci. The lipase activity was visualized as a clear halo surrounding the colonies with the eventual accumulation of an opaque precipitate.

### Data availability.

WGS data for all isolates sequenced in this study have been deposited in the European Nucleotide Archive under study accession PRJEB24782. Individual accession numbers are also included in the Table S2 and Microreact URL https://microreact.org/project/qpaxQtz-9.
