# Supplementary figures and images for: Genomic Epidemiology of CC30 Methicillin-Resistant Staphylococcus aureus Strains from Argentina Reveals Four Major Clades with Distinctive Genetic Features
Source: mSphere. 2021 Mar 10;6(2):e01297-20. doi: 10.1128/mSphere.01297-20 (PMC8546718; doi:10.1128/mSphere.01297-20)

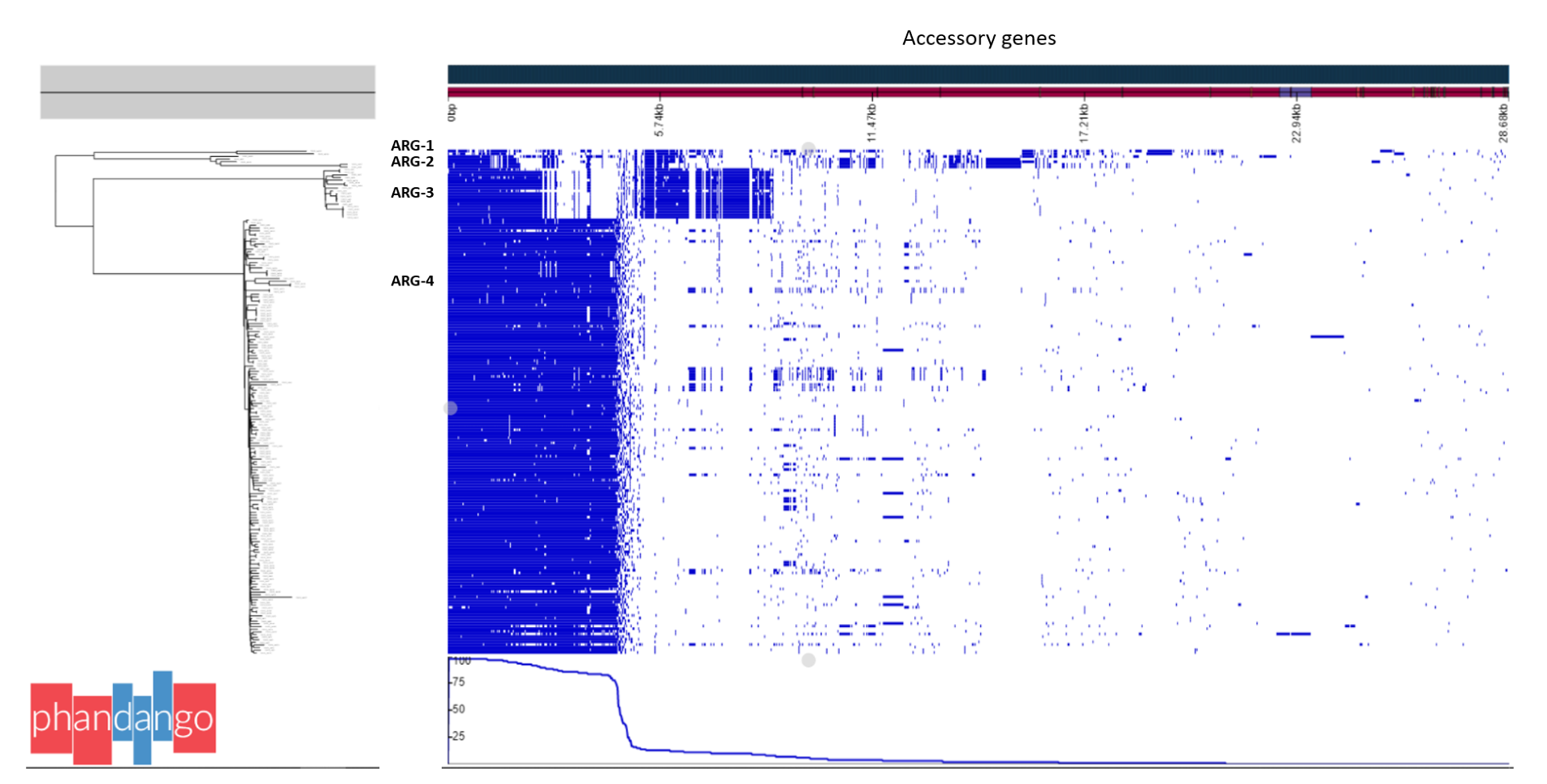

Supplement: FIG S1 [file msphere.01297-20-sf001.tif]

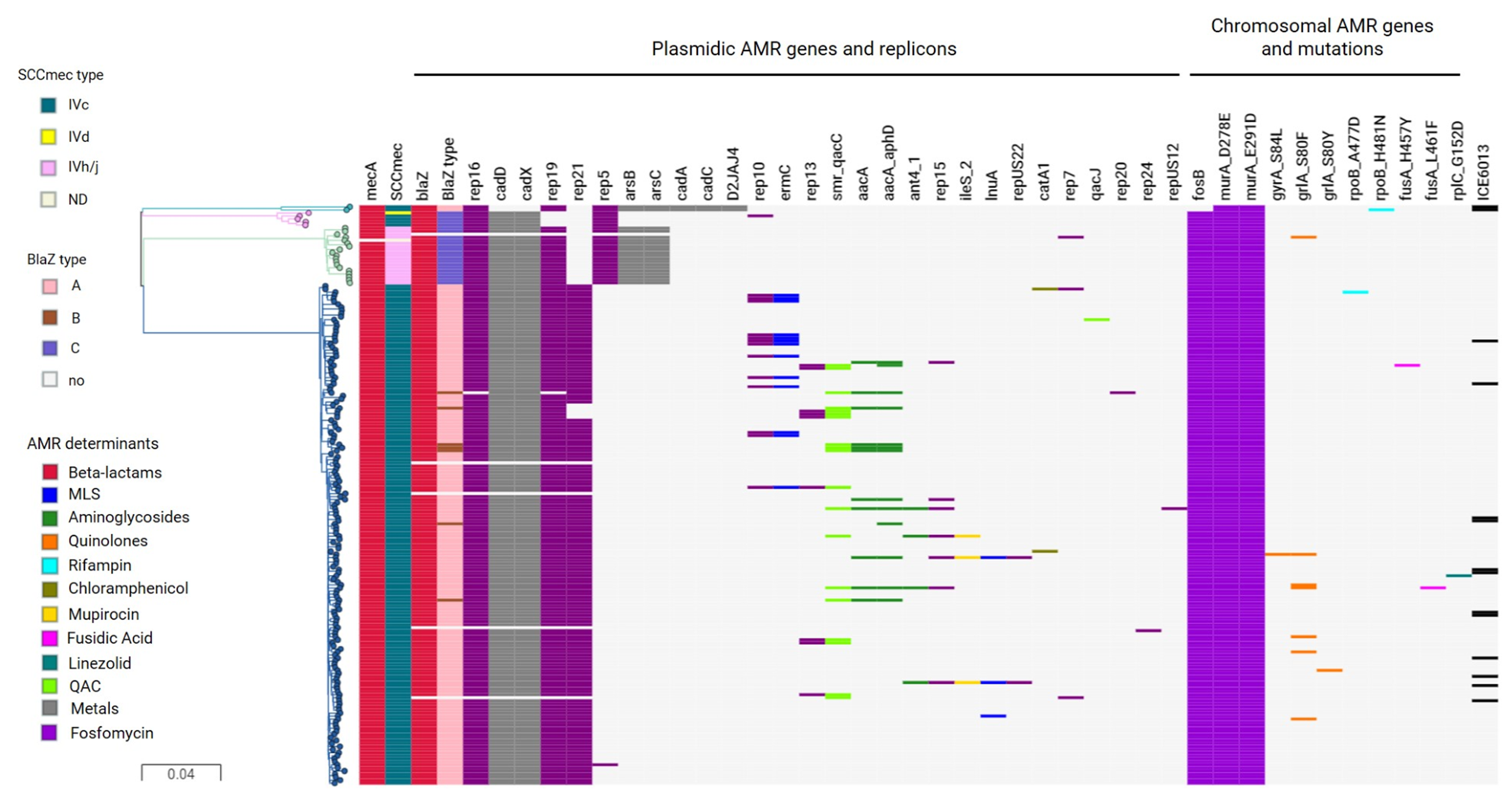

Supplement: FIG S2 [file msphere.01297-20-sf002.tif]

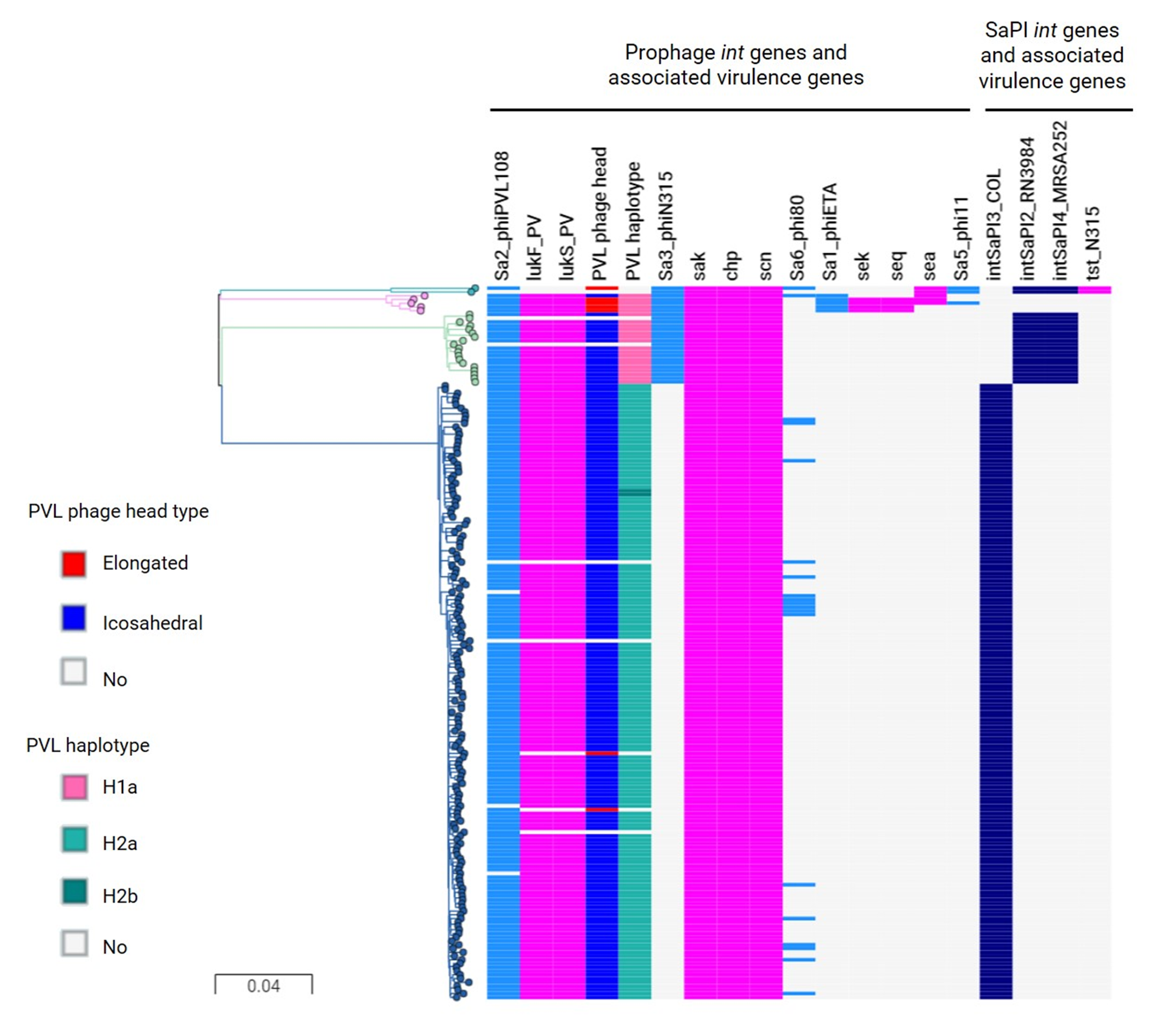

Supplement: FIG S3 [file msphere.01297-20-sf003.tif]

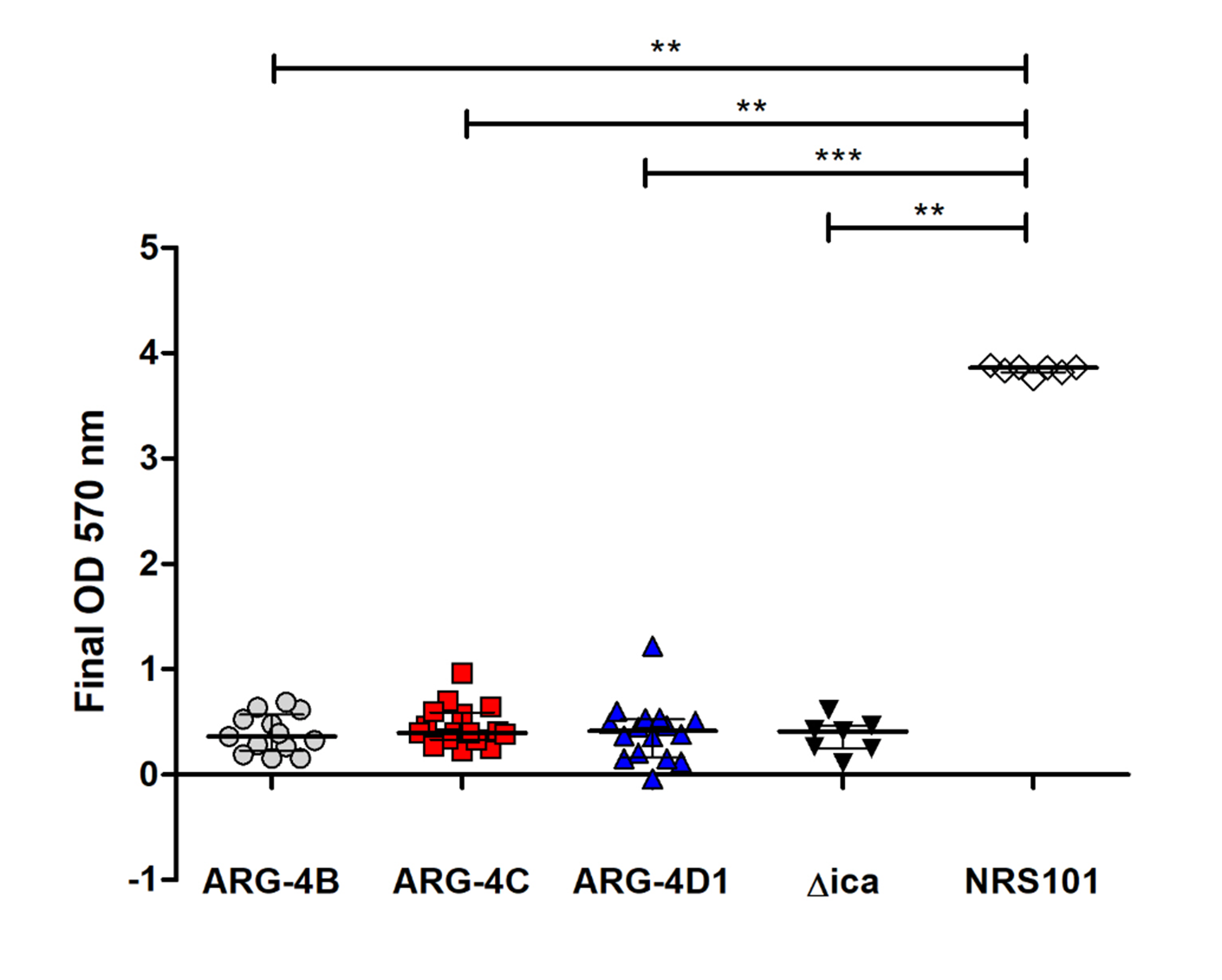

Supplement: FIG S4 [file msphere.01297-20-sf004.tif]

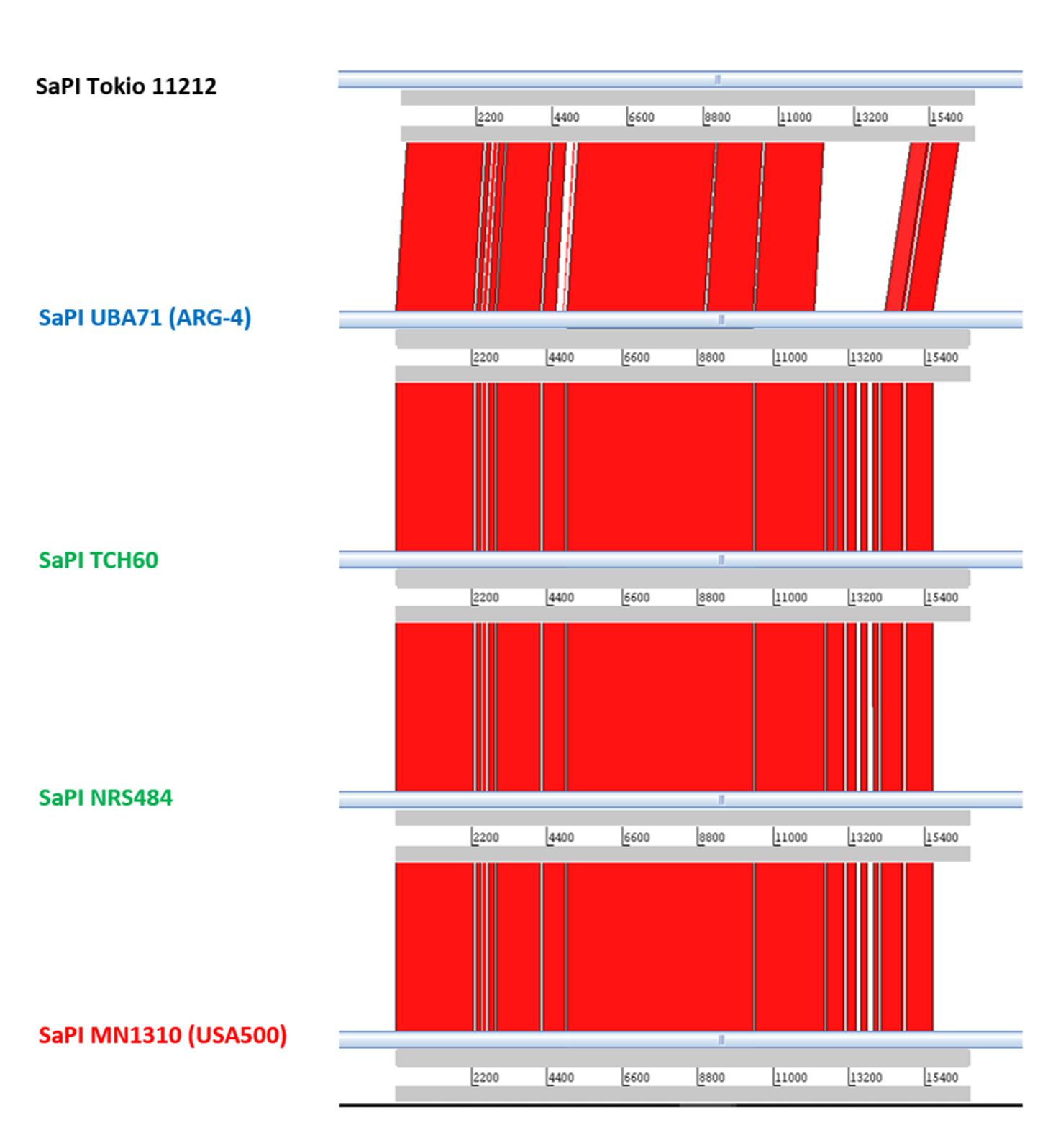

Supplement: FIG S5 [file msphere.01297-20-sf005.tif]
